# Removal of Disinfection By-Products from Contaminated Water Using a Synthetic Goethite Catalyst via Catalytic Ozonation and a Biofiltration System·

**DOI:** 10.3390/ijerph110909325

**Published:** 2014-09-10

**Authors:** Yu-Hsiang Wang, Kuan-Chung Chen

**Affiliations:** 1 Department of Environmental Science and Engineering, National Pingtung University of Science and Technology, Neipu, Pingtung County 912, Taiwan; E-Mail: P9831002@mail.npust.edu.tw; 2 Emerging Compounds Research Center (ECOREC), National Pingtung University of Science and Technology, 1 Shuehfu Rd., Neipu, Pingtung 91201, Taiwan

**Keywords:** catalytic ozonation, goethite, biofiltration, disinfection by-products, emission-excitation matrix, bromine incorporation factor

## Abstract

The effects of synthetic goethite (α-FeOOH) used as the catalyst in catalytic ozonation for the degradation of disinfection by-product (DBP) precursors are investigated. A biofiltration column applied following the catalytic ozonation process is used to evaluate the efficiency of removing DBP precursors via biotreatment. Ozone can rapidly react with aromatic compounds and oxidize organic compounds, resulting in a decrease in the fluorescence intensity of dissolved organic matter (DOM). In addition, catalytic ozonation can break down large organic molecules, which causes a blue shift in the emission-excitation matrix spectra. Water treated with catalytic ozonation is composed of low-molecular structures, including soluble microbial products (SMPs) and other aromatic proteins (APs). The DOM in SMPs and APs is removed by subsequent biofiltration. Catalytic ozonation has a higher removal efficiency for dissolved organic carbon and higher ultraviolet absorbance at 254 nm compared to those of ozonation without a catalyst. The use of catalytic ozonation and subsequent biofiltration leads to a lower DBP formation potential during chlorination compared to that obtained using ozonation and catalytic ozonation alone. Regarding DBP species during chlorination, the bromine incorporation factor (BIF) of trihalomethanes and haloacetic acids increases with increasing catalyst dosage in catalytic ozonation. Moreover, the highest BIF is obtained for catalytic ozonation and subsequent biofiltration.

## 1. Introduction

Disinfection by-products (DBPs) are regulated in many countries, due to their genotoxicity, mutagenicity and carcinogenicity [1]. Trihalomethanes (THMs) and haloacetic acids (HAAs) are two major groups of carbonaceous DBPs (C-DBPs) that form during chlorination in drinking water treatment [1]. Natural organic matter (NOM) generally consists of humic substances in raw water and has been recognized as an important source of DBP precursors [2]. Consequently, developing efficient treatment processes for the removal of NOM and the reduction of DBP formation has received a lot of research interest.

Ozone is a strong oxidant and disinfectant and, thus, is widely used in drinking water treatment. The advantage of using ozone in drinking water treatment is that it can effectively eliminate color and taste, as well as remove DBP precursors (such as humic substances) in water [3]. Ozonation has a few disadvantages that limit its application in water treatment technology. For example, the direct oxidation of organic compounds by ozone is a selective reaction that has slower reaction rate constants compared to those of indirect and nonselective hydroxyl radical oxidation [4]. Moreover, ozone does not completely oxidize dissolved organic matter (DOM) [5]. Fluorescence spectroscopy has been used to analyze water before and after ozonation, with results indicating structural changes in DOM and the formation of a lot of low molecular weight compounds [6,7]. These compounds have been confirmed to be biodegradable organic carbon (BDOC) [5,7], which can cause the regrowth of microorganisms in a drinking water distribution system. Ozonation integrated with biotreatment has been increasingly used in water treatment plants in order to solve the BDOC problem and further improve the removal of DOM and ammonia prior to disinfection [5,8,9,10].

The heterogeneous catalytic ozonation process (HCOP) has recently received a lot of attention in water treatment due to its high effectiveness in the degradation and mineralization of refractory organic pollutants [11,12,13]. Nawrocki and Kasprzyk-Hordern [13] conducted a literature review and reported that catalysts used in catalytic ozonation include metal oxides (e.g., MnO_2_, TiO_2_, Al_2_O_3_ and FeOOH) and metals or metal oxides on metal oxide supports (e.g., ZrO_2_/Al_2_O_3_, CeO_2_/TiO_2_, Cu-Al_2_O_3_, Cu-TiO_2_, TiO_2_/Al_2_O_3_ and Fe_2_O_3_/Al_2_O_3_). They proposed three main mechanisms for the HCOP reaction: (i) direct ozone reacts with surface functional groups on the surface of catalysts, initiating the production of hydroxyl radicals (·OH); (ii) the organic molecules adsorb onto the surface of catalysts and then react with ozone/·OH; and (iii) both ozone and organic molecules are simultaneously adsorbed onto the catalyst surface, and there is a subsequent interaction between the chemisorbed species [13]. The removal efficiency of organic compounds obtained using HCOP is higher than that obtained using ozonation alone [9,11,12,13]. Many studies have demonstrated that the DBP formation potential (DBPFP) in water treated with HCOP is significantly lower compared to that after ozonation alone [9,14]. However, few studies have discussed the structural changes in DOM caused by catalytic ozonation and their influence on DBPFP [6,15]. Another important issue regarding the formation of DBPs is that water containing bromide ion (Br^−^) will influence the species of THM and HAA during chlorination and might cause the formation of bromate after the ozonation treatment [16]. The Br-THMs, such as bromodichloromethane, dibromochloromethane and bromoform, and Br-HAAs, such as bromoacetic acid, bromochloroacetic acid, bromodichloroacetic acid, bromodichloroacetic acid, dibromoacetic acid, chlorodibromoacetic acid and tribromoacetic acid, will be formed in the chlorination of water containing Br. In our previous study [9], the proportions of Br-THMs and Br-HAAs in the HCOP-treated water were about 35%–40% in THMs and 10%–50% in six HAAs (HAA6), respectively. The reaction of chlorine and bromide can cause the formation of hypobromous acid (HOBr) and hypobromite (OBr), which are more powerful than hydrochlorous acid (HOCl). Westerhoff *et al.* [17] demonstrated that HOBr reacts with NOM faster than HOCl, which leads to greater formation of Br-DBPs. Therefore, the ratio of HOBr/HOCl plays an important role in DBP formation. Bromate has been recognized as a potential human carcinogen [1]. However, the effect of HCOP on the formation of bromate is rarely reported.

A previous study used goethite (FeOOH), which is a byproduct released from the corrosion of iron pipes, as a reductant and an adsorbent in drinking water treatment to investigate its influence on DBP formation [18]. They concluded that the structures of Fe (II) (Fe (II) bound to FeOOH or magnetite and aqueous Fe (II)) will influence the degradation rate of DBPs. However, only chloroform (CHCl_3_) and trichloroacetic acid (TCAA) were analyzed in their study. Hassan *et al.* [19] investigated the adsorption of NOM with FeOOH and its effect on chlorine decay. They found that the adsorption of NOM onto the FeOOH caused a change in the character of the NOM, which increased chlorine consumption. The concentration of THMs increased with increases in the FeOOH dosage, but the concentration of five HAAs (HAA5) did not follow the same trend. Their study did not further analyze or identify the change in the DOM structure before and after the adsorption of the FeOOH. Kaplan Bekaroglu *et al.* [20] used FeOOH coated on pumice as an adsorbent to investigate its effect on the removal efficiency of DBP precursors. Their study demonstrated that FeOOH coated on pumice can effectively adsorb dissolved organic carbon (DOC) from raw water that contains mainly hydrophilic and low molecular weight NOM moieties (specific ultraviolet absorbance (SUVA) < 2.0 L·(mg-m)^−1^). High reduction (>85%) of THMs and nine HAAs (HAA9) was obtained through adsorption with 6 g·L^−1^ of iron-coated pumice. They also reported that the bromide concentration (up to 550 µg·L^−1^) did not have an impact on the DOC uptake. With the exception of adsorption on FeOOH, Park *et al.* [21] demonstrated that FeOOH as a catalyst used in HCOP promoted large ·OH formation, which effectively decreased the NOM concentration in the treated water compared to ozonation alone. However, the researchers did not further evaluate the DBPFP in the treated water.

In this study, goethite (α-FeOOH) is used as the catalyst in a fluidized-bed reactor (FBR) for the purpose of catalytic ozonation followed by biofiltration. This system is constructed to investigate the transformation of DOM via emission-excitation matrix (EEM) spectra, to evaluate the removal efficiency of dissolved organic carbon (DOC), ultraviolet absorbance at 254 nm (UV254) and specific ultraviolet absorbance (SUVA) and C-DBP (THMs and HAAs) formation potential during chlorination. The bromine impact factor (BIF) of C-DBPs and bromate concentrations are also investigated in order to study the formation and speciation of DBPs under various operating conditions and treatment processes.

## 2. Materials and Methods

### 2.1. Catalytic Ozonation and Biofiltration

**Figure 1:**
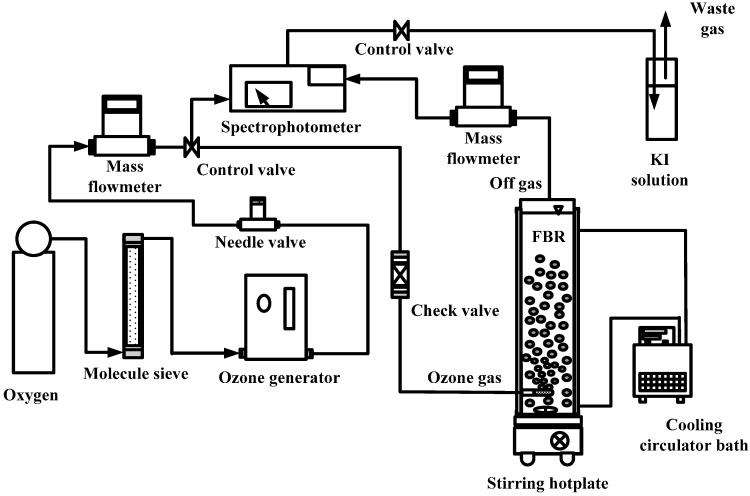
Schematic representation of the catalytic ozonation system.

A catalytic ozonation FBR system in a semi-continuous mode and a biofiltration column were used in this study. A schematic diagram of the catalytic ozonation FBR system is shown in Figure 1. This system comprises a clear acrylic column with a diameter of 6 cm, a height of 38 cm and a volume of 1.04 L. The reaction time was 60 min. The experimental operation parameters are given in Table 1.

The water treated with the FBR system was further treated in a biofiltration column in recirculation mode for 3 days to evaluate the removal efficiency of DBP precursors using the biotreatment process. The biofiltration column from a previous study was adopted [9]. Untreated Wu-Lo River (WLR) water was used to seed the biofiltration column over a period of 4 months. The treatment efficiency of biofiltration was verified to be stable by periodically analyzing the effluent quality.

**Table 1:** Operating conditions for the catalytic ozonation system.

Ozone concentration (mg·L^−1^)	0, 2.5
Gas flow (mL·min^−^^1^)	50
Water temperature (°C)	20
Catalyst type	α-FeOOH
Catalyst dosage (g·L^−^^1^)	0.5, 1.0, 1.5

### 2.2. Catalyst Preparation

**Figure 2:**
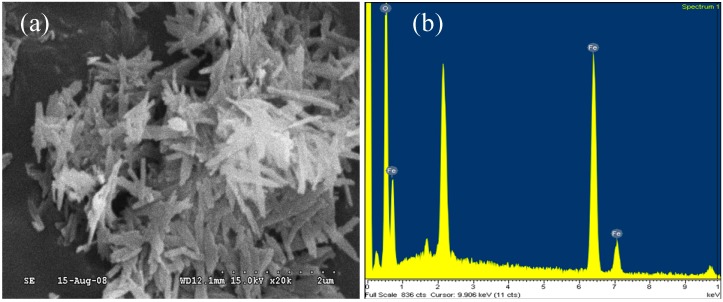
(**a**) SEM image and (**b**) EDS spectrum of the α-FeOOH catalyst.

α-FeOOH catalyst was synthesized following a method developed by Sui *et al.* [22]. A solution of ferric nitrate (Fe(NO_3_)_3_·9H_2_O, 0.25 M) was added to a sodium hydroxide solution (NaOH, 5 N); the pH was adjusted to >12, and the solution was allowed to stand for 48 h. The mixture was filtered through a 0.45-µm filter (C045A047A, Advantec, Tokyo, Japan) to collect the precipitates. The precipitates were then rinsed with distilled water until the effluent remained at a neutral pH. Then, the precipitate was dried at 120 °C for 24 h before being used in the experiments. The catalysts were characterized using scanning electron microscopy (SEM) and energy-dispersive X-ray spectroscopy (EDS) on a Hitachi S-3000N system. The results are shown in Figure 2. The Brunauer–Emmett–Teller (BET, Beckman Coulter SA3100) surface area of the α-FeOOH catalyst was 61.9 m^2^·g^−1^, as determined by N_2_ physisorption at 77 K.

### 2.3. Water Source

Water samples were collected from WLR, Pingtung, Taiwan, which is polluted with domestic and agricultural wastewaters. Water samples were collected in 25-L polyethylene tanks and stored in a refrigerator at 4 °C for less than 7 days. Samples were warmed back to the experimental operating temperature (20 °C) before being used and were passed through a 0.45-µm filter (C045A047A, Advantec, Japan) to remove coarse suspended and colloidal solids.

### 2.4. Fluorescence Measurements

The fluorescence spectra of the water samples were measured and recorded on a fluorophotometer (F-7000, Hitachi, Japan) to determine the characteristics of the organic matter. The excitation-emission matrix (EEM) of each sample was generated by scanning at excitation (ex) wavelengths of 200 to 450 nm in 5-nm steps and by detecting the emitted fluorescence (em) between 250 and 550 nm in 2-nm steps. The scanning speed was 5000 nm·min^−1^. During the course of the fluorescence analysis, the Raman scattering peak intensity for Milli-Q water (excitation at 350 nm, emission at 395–400 nm) was recorded as a standard to verify the instrument stability. Detailed information regarding EEM analysis is available elsewhere [23].

### 2.5. DOC, UV254 and SUVA

The DOC was analyzed in accordance with Standard Method 5310B [24]. A total organic carbon (TOC) analyzer (Model TOC-VCSH, Shimadzu, Tokyo, Japan) was used to determine the non-purgeable organic carbon according to the combustion catalytic oxidation/Non-Dispersive Infrared (NDIR) method. UV254 was analyzed in accordance with Standard Method 5910B [24] using a UV-Vis spectrometer (DR-5000, HACH) at 254 nm, analyzed in triplicate. The SUVA value was calculated as UV254 times 100 divided by the DOC concentration. All water samples were analyzed in triplicate.

### 2.6. Chlorination of the Water Samples

A stock solution of sodium hypochlorite (NaOCl, Fisher Scientific, Pittsburgh, PA, USA) was used for chlorination. It was standardized according to Standard Method 4500B [24]. The water samples were dosed with a chlorine concentration that allowed the free residual chlorine concentration to be in the range of 0.2 to 1.0 mg·L^−^^1^ after 48 h of incubation at room temperature according to the procedures delineated in Standard Method 2350B and Standard Method 5710C [24]. Chlorine residuals were analyzed using the DPD ferrous titrimetric method in Standard Method 4500F [24].

### 2.7. THMs and HAAs

THMs and nine HAAs (HAA9) were analyzed using a gas chromatograph (Agilent HP 6890N) outfitted with a ^63^Ni electron capture detector (ECD), an auto-sampler and a Supelco Equity^TM^-5 column (30 m × 0.25 mm ID). The instrument operating conditions were set according to our previous work [9]. Three replications of THM and HAA9 measurements were performed for each sample.

Four THM compounds, namely chloroform (CHCl_3_), bromodichloromethane (CHBrCl_2_), dibromochloromethane (CHBr_2_Cl) and bromoform (CHBr_3_), were monitored in this study. The THMs were extracted with hexane in accordance with Standard Method 6232B [24].

The HAA9 compounds, namely monochloroacetic acid (MCAA), mono bromoacetic acid (MBAA), dichloroacetic acid (DCAA), bromochloroacetic acid (BCAA), trichloroacetic acid (TCAA), bromodichloroacetic acid (BDCAA), dibromoacetic acid (DBAA), chlorodibromoacetic acid (CDBAA) and tribromoacetic acid (TBAA), were quantified according to the United States Environmental Protection Agency (USPEA) Method 552.2 [25].

### 2.8. Bromide and Bromate

Bromide and bromate samples were analyzed following USEPA Method 300.1 [26] using ion chromatography (Dionex ICS-5000) with conductivity detection. The eluent was 9.0 mM sodium carbonate (Na_2_CO_3_, Merck) and 15 mM NaOH. A pre-column (AG9-HC, Dionex) was connected to an analytical column (AS9-HC, Dionex). All samples were filtered using prerinsed 0.45-µm nylon syringe filters (N045A045A, Advantec, Tokyo, Japan) prior to analysis and were analyzed in triplicate. An injection volume of 100 µL was used. A calibration mixture containing bromide and bromate was prepared from granular ACS (American Chemical Society)-grade reagents.

## 3. Results and Discussion 

### 3.1. EEM Fluorescent Characteristics of DOM

**Figure 3:**
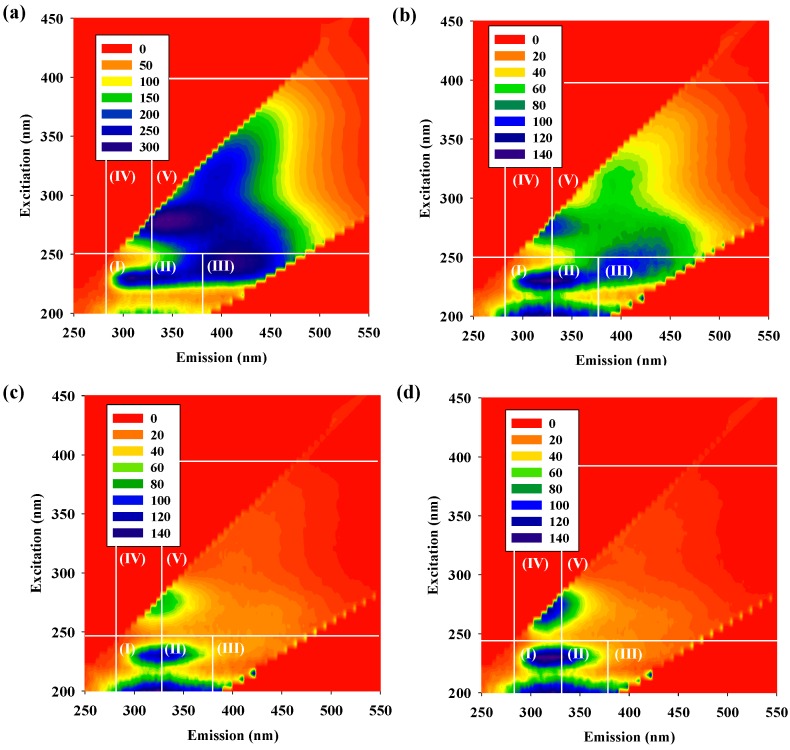
Representative fluorescence EEM spectra of DOM for (**a**) raw water and water treated with (**b**) ozonation, (**c**) catalytic ozonation (α-FeOOH, 0.5 g·L^−^^1^) and (**d**) catalytic ozonation (α-FeOOH, 1.5 g·L^−^^1^).

The EEM spectra of the DOM fractions before and after ozonation and catalytic ozonation with α-FeOOH are shown in Figure 3. In general, the EEM spectra of DOM fractions can be divided into five regions [23]. Region I (ex = 200–250 nm, em (emitted fluorescence) = 280–330 nm) and Region II (ex = 200–250 nm, em = 330–380 nm) are related to simple aromatic proteins (APs). Region III (ex = 200–250 nm, em = 380–550 nm) represents fulvic acid-like materials (FAs). Region IV (ex = 250–400 nm, em = 280–380 nm) and Region V (ex = 250–400 nm, em = 380–550 nm) are related to soluble microbial products and humic acid-like organics, respectively.

As can be seen in Figure 3a, three main compositions of raw water were found in the EEM spectra, namely Regions II, III and IV. Regions II and IV are also associated with soluble microbial products (SMPs) and other APs. Previous studies have reported that SMPs and APs are the most important precursors of nitrogenous DBPs (N-DBPs), because they contain elevated levels of organic nitrogen [27]. FAs (region III) are the main precursors of C-DBPs [7]. The intensity of the fluorescence of the EEM spectra in all five excitation-emission regions decreased in the water treated with the ozonation process (Figure 3b). This result suggests that ozonation can destroy the specific molecule groups (mainly aromatic compounds) and decrease the fluorescence intensity [15]. The normalized excitation-emission area volumes (ϕ_i,n_) [23] of the individual regions in the EEM spectra of the raw and treated water samples are shown in Table 2. It can be seen that the APs (Region II) and SMP substances (Region IV) were the two dominant organic matters after the ozonation of the raw water. This phenomenon can be attributed to the fact that ozone easily attacks the aromatic ring of humic acid-like and fulvic acid-like natural organic matter through an electrophilic reaction, which resulted in the apparent decrease in the fluorescence intensity of Region III and Region V. Unlike the ozonation process, catalytic ozonation can produce ·OH, which is a stronger oxidant than ozone, through the catalytic decomposition of dissolved ozone on the surface of the catalyst [28]. Therefore, the catalytic ozonation of the raw water resulted in a significant reduction in the fluorescence intensity of the DOM fractions in the treated water and led to the blue-shift effect (a shift to the region of short excitation and emission wavelengths) in the EEM spectra (Figure 3c,d). Many researchers have demonstrated that the blue-shift in emission maximum is caused by a reduction in the degree of the π-electron system, such as a decrease in the number of aromatic rings, a reduction of the conjugated bonds in a chain structure or the conversion of a linear ring system to a non-linear system [6,7,29,30]. This suggests that the dissolved ozone molecular and ·OH in HCOP can react with humic acid-like (Region V) and fulvic acid-like (Region III) substances to form oxidation byproducts. Some researchers have also found that the blue-shift in an excitation wavelength corresponds to the relatively small molecular weight of the molecules in the NOM fractions [6,7,31]. Therefore, this phenomenon can be ascribed to the breakdown of large molecules into smaller fragments and a decomposition of aromatic compounds. Some literature has suggested that the hydrophobic NOM fractions will be removed by the HCOP, which will cause an increase in the hydrophilic NOM fractions [6,21,32]. The hydrophobic NOM fraction has a higher SUVA value than other fractions, indicating that it has highly aromatic compounds and that the molecular weight would be, in general, more than 1000 Da [21], whereas the hydrophilic NOM fraction has a relatively low SUVA value, and the molecular weight would be less than 1000 Da [21]. Herein, we would like to mention that the characteristics of DOM are highly dependent on the sampling locations, time of collection, local weather, *etc.*, which are likely to affect the molecular size distribution of DOM. Her *et al.* [33] indicated that DOM composition is different between various water sources, which results in different EEM spectra after molecular size fractionation. Therefore, the interpretation of data regarding the EEM spectra of DOM characterization in different water sources should be made with great care.

The fluorescence index (FI), which is defined as the ratio of emission intensity at 450 and 500 nm for emission spectra measured at a excitation wavelength of 370 nm in EEM spectra [15,34], of the individual region in the EEM spectra of the raw and treated water samples is also shown in Table 2. McKnight *et al.* [34] found that DOM aromaticity is inversely correlated with the FI. Rodríguez *et al.* [15] observed that highly aromatic compounds are present in higher molecular weight (>1000 Da by molecular size fractionation) fractions. As can be seen in Table 2, the FI of the treated water increased with increases in the catalyst loading in the HCOP. This result implies that the breakdown of large molecules occurred during the HCOP.

**Figure 4:**
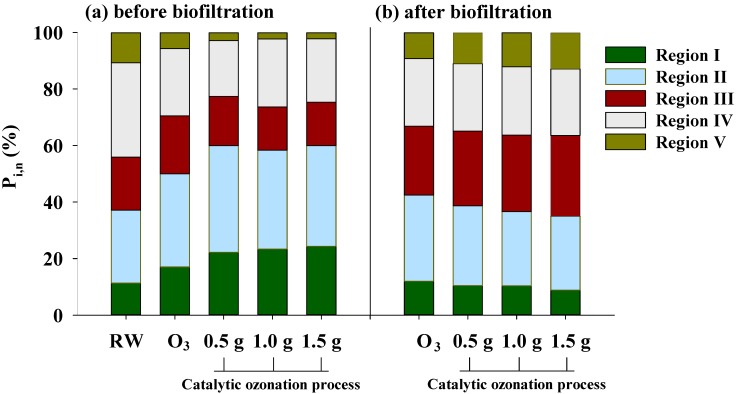
Changes in the P_i,n_ of the DOM samples after catalytic ozonation and catalytic ozonation/biofiltration (RW: raw water; O_3_, ozonation; ozone concentration: 2.5 mg·L^−1;^ gas flow: 50 mL·min^−1;^).

Furthermore, the fluorescence regional integration (FRI) technique developed by Chen *et al.* [23], which calculates the integration of the volume beneath each EEM region, was used to evaluate the quantity of each DOC fraction. The percent fluorescence response (P_i,n_) for each EEM region was calculated based on the FRI technique. The results are shown in Figure 4. P_i,n_ represents the composition of DOM and its distribution in the five excitation-emission regions. As shown in Figure 4a, the three main compositions of DOM in the raw water were Region IV (33.8%), Region II (26.1%) and Region III (19.0%). The percentage of the low-molecular structures (the sum of Regions I and II) in the water treated with catalytic ozonation increased with increases in the catalyst dosage, becoming the main composition of the DOM. Figure 4b shows the P_i,n_ of water treated with biofiltration following either ozonation or catalytic ozonation. It can be seen that the percentage of simple APs (Region I) and SMPs (Region II) decreased compared to that for ozonation or catalytic ozonation alone (Figure 4a). This implies that these substances can be preferentially removed by biofiltration due to their high biodegradability [10]. The percentage of low-molecular structures (sum of Regions I and II) also decreased with increases in the catalyst dosage for dosages of 0 to 1.5 g·L^−1^. This result indicates that the α-FeOOH catalyst in catalytic ozonation can promote the formation of BDOC, which can be degraded by the subsequent biofiltration.

**Table 2:** Normalized excitation-emission area volumes (ϕ_i,n_ × 10^7^) and fluorescence index (FI) of the individual regions in the EEM spectra. FI, fluorescence index.

	Region I (×10^7^)	Region II (×10^7^)	Region III (×10^7^)	Region IV (×10^7^)	Region V (×10^7^)	FI
Raw water	4.31	9.83	7.14	12.7	4.09	2.19
Ozonation	3.29	6.35	3.95	4.59	1.11	2.60
HCOP-0.5 g	2.96	5.02	2.33	2.63	3.87	2.69
HCOP-1.0 g	3.31	4.97	2.17	3.15	3.32	2.75
HCOP-1.5 g	3.42	5.00	2.15	3.42	3.16	2.83
Ozonation/Bio.	2.89	7.33	5.87	5.76	2.24	2.05
HCOP-0.5 g/Bio.	3.15	8.49	7.98	7.19	3.34	1.79
HCOP-1.0 g/Bio.	3.87	9.75	10.1	8.97	4.54	1.76
HCOP-1.5 g/Bio.	3.14	9.28	10.1	8.34	4.60	1.72

HCOP, heterogeneous catalytic ozonation process; Bio., biofiltration.

### 3.2. Effects of Catalytic Ozonation and Subsequent Biofiltration on Treated Water Quality

**Figure 5:**
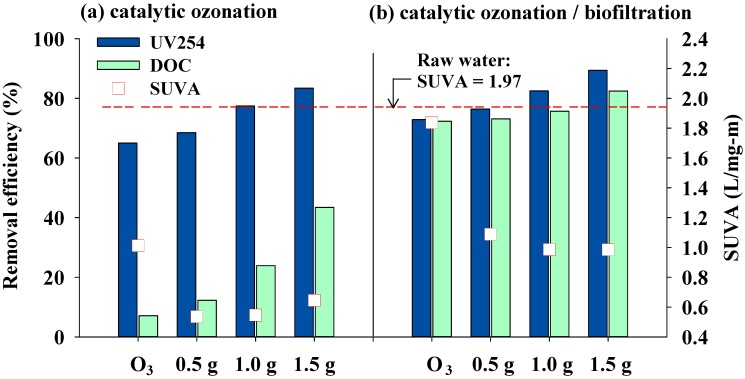
Removal efficiency of DOC and UV254 with various catalyst dosages for (**a**) catalytic ozonation alone and (**b**) catalytic ozonation followed by biofiltration (ozone concentration: 2.5 mg·L^−1^; gas flow: 50 mL·min^−1^).

The concentrations of water quality parameters and DBPs in different experimental conditions are summarized in Table 3. The removal efficiencies of DOC and UV254 for catalytic ozonation and catalytic ozonation followed by biofiltration are shown in Figure 5a,b, respectively. As can be seen in Figure 5a, the removal percentages of DOC for catalyst dosages of 0.5, 1.0 and 1.5 g·L^−1^ are 12%, 24% and 43%, respectively. The removal efficiencies of UV254 for these dosages are 69%, 77% and 83%, respectively. The removal efficiencies for DOC and UV254 both increased with increases in the catalyst dosage. Moreover, the removal efficiency of UV254 was higher than that of DOC during catalytic ozonation. This result reveals that only some DOC was oxidized to carbon dioxide. According to the EEM spectra, the DOM in the WLR raw water is abundant in FAs (Region III). It has been recognized that aromatic groups, which are susceptible to electrophilic attack, are the major structure in fulvic acids. Thus, ozone molecules are very effective in breaking the aromatic structure and, thus, rapidly decrease UV254 absorbance. Compared with ozone, which reacts with organic matter through a selective reaction mechanism, ·OH is a stronger and nonselective oxidant that reacts with organic contaminants quickly and effectively. It was found that catalytic ozonation has a better removal efficiency of UV254 than that obtained using ozonation alone. The removal percentage of UV254 increased with increases in the catalyst dosage in catalytic ozonation. Catalytic ozonation also has a higher DOC removal efficiency in comparison with that obtained using ozonation alone. This result is attributed to the increased formation of ·OH under catalytic ozonation [13,35].

The water treated with ozonation and catalytic ozonation was further treated using biofiltration in recirculation mode for three days (Figure 5b). The results show that the subsequent biofiltration significantly increased the removal efficiencies of DOC (72%–82%) and UV254 (72%–89%) compared to those obtained using catalytic ozonation alone. The results imply that catalytic ozonation can effectively oxidize organic matter and increase the biodegradability of DOC, which is then easily removed by the subsequent biofiltration process. It was also found that the removal efficiencies of DOC and UV254 have a positive correlation with catalyst dosage in catalytic ozonation, which confirms the findings of other studies [5,9].

**Table 3:** Concentrations of water quality parameters and disinfection by-product formation potential (DBPFP) in different experimental conditions. UV254, ultraviolet absorbance at 254 nm; THM, Trihalomethane; HAA, haloacetic acid.

	DOC	UV254	THMFP	HAAFP
	(mg·L^−1^)	(cm^−1^)	(µg·L^−1^)	(µg·L^−1^)
Raw water	9.21 ± 1.6	0.181 ± 0.036	233.5 ± 28.1	535.1 ± 23.4
Ozonation	8.5 ± 0.15	0.062 ± 0.001	179.3 ± 6.7	382.5 ± 4.6
HCOP-0.5	8.0 ± 0.11	0.056 ± 0.002	147.0 ± 8.4	350.5 ± 7.7
HCOP-1.0	7.0 ± 0.14	0.040 ± 0.000	143.0 ± 6.5	246.2 ± 6.4
HCOP-1.5	5.2 ± 0.11	0.030 ± 0.001	102.7 ± 4.1	218.1 ± 8.9
Ozonation/Bio.	2.5 ± 0.15	0.048 ± 0.001	125.0 ± 7.9	112.2 ± 4.5
HCOP-0.5/Bio.	2.5 ± 0.12	0.042 ± 0.003	60.0 ± 8.8	68.3 ± 6.8
HCOP-1.0/Bio.	2.2 ± 0.13	0.031 ± 0.002	43.2 ± 7.2	66.1 ± 6.6
HCOP-1.5/Bio.	1.6 ± 0.11	0.019 ± 0.003	38.7 ± 4.2	64.0 ± 4.7

HCOP, heterogeneous catalytic ozonation process; Bio., biofiltration.

From Figure 5, it can be seen that the water treated with the catalytic ozonation process has lower SUVA values than those for the ozonation process. SUVA has been found to positively correlate with C-DBP concentration during the chlorination of water [36,37]. However, the SUVA values in all of the samples of treated water increased after subsequent biofiltration. This is mainly due to the removal percentage of DOC being much higher than that of UV254 after biofiltration, as can be found by comparing Figure 5a,b. Moreover, soluble microbial products (SMPs) were likely to be released as the biofiltration was operated for three days in recirculation mode. It is reasonable to assume that some SMPs might be released into the treated water, which resulted in the higher SUVA of catalytic ozonation/biofiltration as compared to that of catalytic ozonation.

### 3.3. Effects of Catalytic Ozonation and Subsequent Biofiltration on DBP Formation 

**Figure 6:**
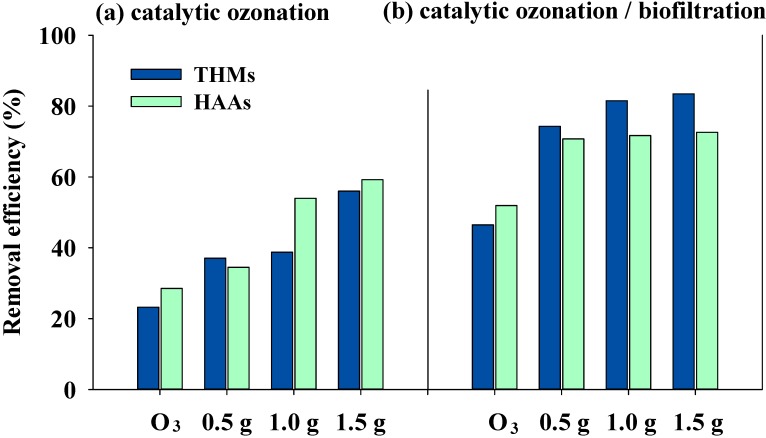
Removal efficiencies of THMs and HAAs with various catalyst dosages for (**a**) catalytic ozonation alone and (**b**) catalytic ozonation followed by biofiltration (ozone concentration: 2.5 mg·L^−1^; gas flow: 50 mL·min^−1^).

The THM and HAA9 formation potential were measured for both the raw water and the water treated with catalytic ozonation and subsequent biofiltration. The experimental results are shown in Figure 6. In the chlorination of raw water, the HAA9 formation potential was higher than the THM formation potential (Table 3). Many researchers have demonstrated that hydrophobic acids, such as humic acids and fulvic acids, are the main THM precursors [3,25]. For the tested WLR raw water, the SUVA value was below 4 mg^−1^·m^−1^, which implies that the DOM in the raw water mainly consisted of hydrophilic acids [3,38,39]. Zhang *et al.* [36] found that hydrophilic acid reacting with chlorine increases the HAA concentration more than it does the THM concentration. This could explain why the HAA9 formation potential was higher than the THM formation potential.

Figure 6 shows the influence of catalyst dosage on the removal efficiency of the THM and HAA9 precursors during catalytic ozonation and the subsequent filtration. The results show that ozonation only decreased 23% and 28% of the formation potential of THMs and HAA9, respectively. With the α-FeOOH catalyst added and the catalyst dosage increased from 0.5 to 1.5 g·L^−1^, the removal efficiencies of THM and HAA9 precursors significantly increased from 37% to 56% and from 35% to 59%, respectively. These results indicate that the DBPFP can be decreased by increasing the catalyst dosage in the catalytic ozonation process. Many researchers have reported that an increase in the catalyst concentration benefits the degradation of organic substances [40,41]. This is due to the formation of highly reactive ·OH in catalytic ozonation and the adsorption of those organic substances onto the catalyst surface [13,41]. Therefore, it is likely that the catalytic activity and adsorption capability of catalysts play an important role in the catalytic ozonation process.

The removal efficiency of DBP precursors for catalytic ozonation followed by biofiltration was also evaluated (Figure 6b). The results show that the removal efficiencies of the THM and HAA9 precursors were 46%–83% and 52%–73%, respectively. This indicates that biofiltration can further remove the THMs and HAA9 precursors after ozonation and catalytic ozonation, which decreases DBP formation during post-chlorination [9,42]. Catalytic ozonation followed by biofiltration significantly improved the removal efficiency of the THM and HAA9 precursors as compared to that of ozonation alone. However, the removal efficiencies of the THM and HAA9 precursors slightly increased when the catalyst dosage was increased from 0.5 to 1.5 g·L^−^^1^ after biofiltration. This result implies that catalytic ozonation not only decreased the formation potential of THMs and HAA9, but also promoted their removal efficiency in the subsequent biofiltration. This observation is in agreement with the findings reported by other researchers, who used a combination of catalytic ozonation and biotreatment [9,43,44].

The concentrations of individual THM and HAA species are presented in Table 4 and Table 5, respectively. A positive correlation (*p* < 0.0005) exists between DBPs in treated water and catalyst dosages. The HCOP followed by biofiltration effectively lowered the concentration of THMs and HAA9. In the case of the concentrations of individual THM species in different experimental conditions (see Table 4), it can be found that the THM species in the treated HCOP water tended to increase the concentration of the Br-THMs. This phenomenon can be attributed to the organic matters being decreased by the HCOP, which, in turn, decreased the reaction between the aromatic group and HOCl and the formation of Cl-THMs. Since the HOBr reacts with NOM faster than HOCl, a greater amount of Br-DBPs formed during the HCOP [17]. The concentration of THMs significantly decreased as the catalyst dosage was increased after being treated using HCOP or HCOP/biofiltration. In addition, the bromide ion in the raw water was at a low concentration, which resulted in the limitation of Br-THMs formation. The DCAA and the TCAA were the two dominant compounds in HAA9 under all tested experimental conditions (see Table 5). The concentration of HAA9 also significantly decreased as the catalyst dosage was increased after being treated by HCOP or HCOP/biofiltration. Regarding the concentration of individual HAA species in chlorinated treated water, the Br-HAAs followed the same trend as the Br-THMs. The concentration of MBAA and BDCAA were apparently increased after the HCOP. This result can be ascribed to the fact that the structure of the degraded organic matters was more likely to react with HOBr during the HCOP. According to our experimental results, it can be concluded that catalytic ozonation followed by biofiltration can produce lower concentrations of THMs and HAA9 during chlorination as compared to those obtained using ozonation alone and catalytic ozonation alone.

**Table 4:** The concentrations of individual THM species under different experimental conditions.

	CHCl_3_ (µg·L^−1^)	CHBrCl_2_ (µg·L^−1^)	CHBr_2_Cl (µg·L^−1^)	CHBr_3_ (µg·L^−1^)	THMs (µg·L^−1^)
Raw water	215.07	18.45	0	0	233.52
Ozonation	152.38	23.31	3.59	0	179.27
HCOP-0.5	95.52	39.68	8.82	2.94	146.95
HCOP-1.0	90.08	35.75	11.44	5.72	142.99
HCOP-1.5	59.59	22.60	12.33	8.22	102.73
Ozonation/Bio.	68.75	41.25	12.50	2.50	125.00
HCOP-0.5/Bio.	30.62	14.41	10.21	4.80	60.03
HCOP-1.0/Bio.	19.02	10.80	8.64	4.75	43.22
HCOP-1.5/Bio.	14.69	10.05	8.51	5.41	38.66

HCOP, heterogeneous catalytic ozonation process; Bio., biofiltration.

**Table 5:** The concentrations of individual HAA species under different experimental conditions.

	MCAA (µg·L^−1^)	MBAA (µg·L^−1^)	DCAA (µg·L^−1^)	TCAA (µg·L^−1^)	BCAA (µg·L^−1^)	BDCAA (µg·L^−1^)	DBAA (µg·L^−1^)	CDBAA (µg·L^−1^)	TBAA (µg·L^−1^)	HAA9 (µg·L^−1^)
Raw water	50.84	7.49	267.57	150.91	13.38	20.87	11.77	6.96	5.35	535.15
Ozonation	35.96	8.42	183.61	99.46	11.48	18.74	12.24	6.89	5.74	382.53
HCOP-0.5	32.59	14.72	132.48	89.02	21.03	31.54	14.02	8.76	6.31	350.48
HCOP-1.0	19.70	14.77	76.33	49.25	19.21	39.40	14.77	7.88	4.92	246.24
HCOP-1.5	16.14	16.36	63.26	41.45	16.58	39.27	13.09	7.64	4.36	218.15
Ozonation/Bio.	18.01	12.86	113.71	56.60	20.58	18.01	12.86	2.06	2.57	257.27
HCOP-0.5/Bio.	9.39	12.06	94.67	32.88	12.84	10.65	10.18	2.04	1.88	156.58
HCOP-1.0/Bio.	8.78	12.27	60.60	30.61	12.58	10.91	11.82	2.27	1.67	151.52
HCOP-1.5/Bio.	7.33	12.90	54.24	27.85	12.17	13.49	13.19	3.23	2.20	146.59

HCOP, heterogeneous catalytic ozonation process; Bio., biofiltration.

### 3.4. Bromine Incorporation Factor and Bromate Concentration

To assess the extent of bromine substitution in DBP when using chlorine as the disinfectant, the BIF was calculated as:

(1) $$\text{BIF}=\frac{\text{DBP}-\text{Br }(\text{µmol}\cdot {\text{L}}^{-1})}{\text{DBP }(\text{µmol}\cdot {\text{L}}^{-1})}\;$$

where DBP-Br is the sum of the molar concentration of bromine incorporated in THMs and HAA9, and DBP represents the molar concentration of THMs or HAA9. The BIF value ranged from zero to three [45,46].

**Figure 7:**
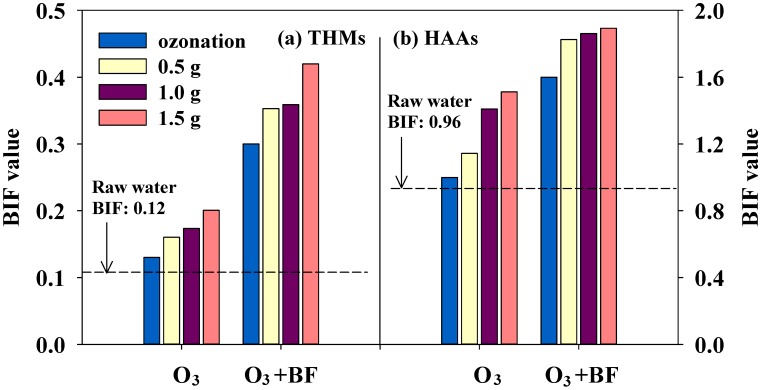
Bromine incorporation factor (BIF) values for (**a**) THMs and (**b**) HAAs for various treatment processes (Raw, raw water; O_3_, catalytic ozonation alone; O_3_ + Bio., catalytic ozonation followed by biofiltration; ozone concentration: 2.5 mg·L^−1^; gas flow: 50 mL·min^−1^).

Figure 7 shows the effect of BIF at various catalyst dosages on catalytic ozonation and subsequent biofiltration. For the THM formation potential in the chlorinated raw water, chloroform (92.1%) was the dominant species. DCAA (50.8%) and TCAA (28.2%) were the two dominant species in HAA9. These results are attributed to the effect of low-concentration bromide in the tested waters [47]. The DCAA concentration was higher than the TCAA concentration in all treated water due to the use of a low chlorine dose in the chlorination [48]. The calculated BIF values of raw water for THMs and HAA9 were 0.12 and 0.96, respectively. After catalytic ozonation, the BIF values for THMs and HAA9 increased from 0.16 to 0.20 and 1.14 to 1.51, respectively, when the catalyst dosage was increased from 0.5 to 1.5 g·L^−1^. During the chlorination of water samples, bromide can be oxidized by chlorine to form hypobromous acid (HOBr) (Equation (2) [37]), which can react with NOM to preferentially produce brominated DBP (Equation (3) [37]) over chlorinated DBP (Equation (4) [37]).

(2) $${\text{Br}}^{-}+\text{HOCl}\to \text{HOBr}+{\text{Cl}}^{-},\;k_{1}=2950{\text{M}}^{-1}\cdot {\text{S}}^{-1}\;$$

(3) $$\text{HOBr}+\text{NOM}\to \text{TOBr},\;k_{2,\text{ pseudo}}=0.2\times 10^{-3}-1.3\times 10^{-3}{\text{ s}}^{-1}\text{ for NOM }$$

(4) $$\text{HOCl}+\text{NOM}\to \text{TOCl},\;k_{3,\text{ pseudo}}=2\times 10^{-5}-3\times 10^{-5}{\text{ s}}^{-1}\text{ for NOM }$$

Many researchers have demonstrated that many parameters can directly influence brominated DBP formation in chlorination, including pH and the ratios of Br^−^/DOC and Br^−^/Cl_2_. However, chlorine can be quickly consumed by NOM, resulting in less chlorine available for chlorine-bromide reactions when water samples contain high DOC concentrations and low bromide concentrations. This can lead to a decrease in the brominated DBP formation. In contrast, the formation of brominated THMs and HAA9 in treated water will increase if water samples are treated using catalytic ozonation to eliminate the DOC before chlorination. The subsequent use of biofiltration increased the BIF values as compared to those for ozonation alone and catalytic ozonation alone. The BIF value and catalyst dosage were also found to be directly proportional. The BIF values for THMs and HAA9 were in the range of 0.30–0.42 and 1.60–1.89, respectively. In our previous study [9], it was found that the number of bromine-containing DBP species increased after catalytic ozonation and biofiltration treatment. The levels of DBAA and BCAA increased with increases in the catalyst dosage, both with and without subsequent biofiltration.

A trace concentration of bromide (28–31 µg·L^−1^) was detected in the WLR raw water. This low concentration of bromide is insufficient for the chemical reaction to form bromate during ozonation in the treated water, even though the rate constants for Br^−^ and HOBr/OBr^−^ oxidation (*k* = 1.1 × 10^9^ M^−1^·s^−1^, *k* = 2 × 10^9^ and 4.5 × 10^9^ M^−1^·s^−1^, respectively) with ·OH are high [49]. Thus, bromate was not detected in any treated water sample in this study. This agrees with the findings of Kingsbury * et al.* [50], whose study found that a low concentration of bromide (<40 µg·L^−1^) in raw water resulted in no significant bromate formation during ozonation.

## 4. Conclusions

The effects of α-FeOOH catalyst used in catalytic ozonation on the structural changes of DOM, water quality parameters (DOC and UV254) and DBP formation potential (THMs and HAA9) were investigated. Catalytic ozonation can break down large organic molecules, change the DOM composition and produce SMPs and APs in the treated water. Subsequent biofiltration can preferentially remove these low molecular weight compounds. The removal efficiencies of DOC and UV254 increased with increases in the catalyst dosage in catalytic ozonation. When a biofiltration unit was installed downstream of the catalytic ozonation, better water quality was obtained in terms of the DOC level and UV254 value compared to those obtained using catalytic ozonation alone. Lower THM and HAA9 formation potentials were obtained by catalytic ozonation and subsequent biofiltration. In the case of the DBP species during chlorination, the BIF values for THM and HAA9 increased for catalytic ozonation and subsequent biofiltration compared to those of the catalytic ozonation. No bromate was detected in the treated water for any treatment process, due to the low bromine concentration in the WLR water.
